# Supplementary material for: Asynchronous magnetic resonance elastography: Shear wave speed reconstruction using noise correlation of incoherent waves
Source: Magn Reson Med. 2022 Oct 27;89(3):990–1001. doi: 10.1002/mrm.29502 (PMC9792433; doi:10.1002/mrm.29502)
Supplement: Supplementary file 1 — DATA S1 MATLAB code used to generate the numerical simulations of Figure 1 [file MRM-89-990-s002.zip › k-Wave/helpfiles/example_ewp_plane_wave_absorption.html]

Plane Wave Absorption Example (k-Wave)


# Plane Wave Absorption Example

This example describes the characteristics of the Kelvin-Voigt absorption model used in the k-Wave simulation functions `pstdElastic2D`, `pstdElastic3D`. It builds on the Explosive Source In A Layered Medium and Modelling Power Law Absorption examples.

- Open the file in the MATLAB Editor
- Run the file in MATLAB

## Contents

- Kelvin-Voigt absorption model
- Absorption of plane waves

## Kelvin-Voigt absorption model

The functions in k-Wave for simulating wave propagation in solid media (`pstdElastic2D`, `pstdElastic3D`) are based on the classical Kelvin-Voigt model of viscoelasticity. For low frequencies and small absorption coefficients, this gives acoustic absorption that is proportional to frequency squared. The compressional and shear absorption coefficients are defined based on this low frequency limit, and are specified in units of dB/(MHz^2 cm).

```
% set the absorption properties
medium.alpha_coeff_compression = 1;     % [dB/(MHz^2 cm)]
medium.alpha_coeff_shear       = 1;     % [dB/(MHz^2 cm)]
```

These absorption coefficients are related to the compressional and shear viscosities of the medium by:

```
alpha_coeff_compression_np = (chi + 2*eta) / (2 * rho0 * cp^3);
alpha_coeff_shear_np       = (eta) / (2 * rho0 * cs^3);
```

where `chi` and `eta` are the compressional and shear viscosity coefficients, respectively, `cp` and `cs` are the compressional and shear sound speeds, respectively, `rho0` is the mass density, and the absorption coefficients are given in units of Nepers/((rad/s)^2 m) (see `db2neper` and `neper2db`). Within the simulation functions, the absorption coefficients are used to calculate the viscosities, and it is these variables that are used in the model. The absorption coefficients can be defined either as a single value, or as a matrix the same size as the computational grid.

## Absorption of plane waves

In this example, the absorption of compressional and shear waves is demonstrated by generating plane waves using a velocity source. A compressional plane wave can be generated by assigning a velocity source parallel with the direction of wave propagation, while a shear plane wave can be generated by assigning a velocity source in the perpendicular direction. In both case, the PML must be switch off in the direction perpendicular to the direction of the wave by setting the PML absorption to zero (see Controlling The Absorbing Boundary Layer).

The absorption encapsulated by the model is extracted using the velocity traces recorded at two sensor points separated by some distance (see Modelling Power Law Absorption). The time traces and absorption behaviour are shown in the figure below. The modelled absorption is shown with the open circles, while the corresponding analytical frequency power law is shown with the solid line. There is a very close agreement up to the Nyquist limit of two points per wavelength, illustrating that the desired absorption behaviour is captured by the model.
